# Barriers to breast self examination practice among Malaysian female students: a cross sectional study

**DOI:** 10.1186/s40064-015-1491-8

**Published:** 2015-11-11

**Authors:** Mehrnoosh Akhtari-Zavare, Muhamad Hanafiah Juni, Irmi Zarina Ismail, Salmiah Md Said, Latiffah A. Latiff

**Affiliations:** Cancer Resource and Education Center, Universiti Putra Malaysia, 43400 Serdang, Selangor Malaysia; Department of Community Health, Faculty of Medicine and Health Science, Universiti Putra Malaysia, 43400 Serdang, Selangor Malaysia; Department of Family Medicine, Faculty of Medicine and Health Science, Universiti Putra Malaysia, 43400 Serdang, Selangor Malaysia

**Keywords:** Breast cancer, Breast self-examination, Barriers, Young female, Malaysia

## Abstract

**Introduction:**

Breast cancer is the most frequent cancer and the second reason of cancer deaths among woman worldwide, including Malaysia. The objective of this paper is to assess the practice of breast self-examination (BSE) and identify the barriers of BSE practice among undergraduate female students in Malaysia.

**Methods:**

A cross-sectional study conducted among 810 female undergraduate students in Klang Valley, Malaysia between April–Jun 2012. Data was collected via self-administered questionnaire which was developed and pre-tested for this study.

**Results:**

The majority of respondents were Malay 709 (95.6 %) and single 719 (96.9 %) with a mean age of 21.7 (1.1). Only hundred eleven (15 %) of the participants had a family history of breast cancer. 70.5 % of the respondents do not practice breast self-examination, 70.5 % do not know how to do it, 64.7 and 61.5 % reported no symptoms of breast cancer and worries to detect breast cancer, respectively. Univariate analysis showed that age, marital status and personal history of breast disease were statistically associated with the practice of breast self-examination.

**Conclusion:**

In this study, a high percentage of respondents were aware of breast cancer but do not perform breast self-examination. Knowledge, socio-cultural and environmental factors were identified as barriers; so it is recommended that knowledge among the public about breast cancer and promotion of public breast health awareness campaigns through the media should be carried out.

## Background

Nowadays, breast cancer is one of the most frequently detected cancers and is the major cause of death among women worldwide (Loh and Chew 2011). Based on the National Cancer Registries in Asian countries, variation for crude incidence rate of breast cancer is 21.3 per 100,000 population in Jordan (Petro-Nustus and Mikhail 2002), 21.4 per 100,000 in Iran (Sadjadi et al. 2009), 24.1 per 100,000 in Turkey (Nahcivan and Secginli 2007) and 52 per 100,000 population in Japan and South Korea (Youlden et al. 2014). Malaysia being one of the countries in the Asian region affected with modernization is heavily burdened with the problem, with breast cancer occurring in 34.86 per 100,000 population (Hisham and Yip 2004).

In Malaysia, breast cancer is the most common cancer and the first cause of death from cancer among women. According to the National Cancer Registry, there were 3525 female breast cancer cases registered in Malaysia, and 1 out every 19 Malaysian women has the chance of getting breast cancer during their lifetime (National Cancer Registry 2006).

Early detection of breast cancer can reduce its morbidity and mortality (Avci 2008). Mammography, clinical breast examination and breast self-examination (BSE) are considered effective methods for early detection of breast cancer (Avci 2008). There are arguments surrounding the efficacy of BSE. A large well-conducted randomised controlled trial among 266,064 textile workers in Shanghai which teaching women to perform BSE showed distribution of tumor size and stage at diagnosis as well as mortality rate for breast cancer was nearly same between women in the two arms of the trial (Thomas et al. 2002). With these result, numerous organisations including US Preventive Service Task Force (2009), the Canadian Task Force on Preventive Health Care (Baxter 2001) determined that breast self-examinations no more benefit for women.

Although BSE alone is not sufficient for early detection of breast cancer, but it is still an important screening tool for early detection of breast cancer in developing countries, because it is cheap, widely available, and does not require complex technical training (Giridhara et al. 2011). Overall, by performing regular BSE, women familiar with the structure of normal breasts will be motivated to attend screening clinics for mammography and clinical breast examination (Giridhara et al. 2011; Tavafian et al. 2009).

The Malaysian Ministry of Health has been promoting BSE and annual breast examinations by trained health workers as part of breast health awareness campaigns since 1995 (Ministry of Health Malaysia 1999). While the majority of women seem to be aware of breast self-examinations, yet many still do not know how to perform it properly (Soyer et al. 2007). Studies conducted among different groups of women in Malaysia showed that monthly BSE practice ranged from 19.6 to 36.7 %, respectively (Akhtari-Zavare et al. 2015; Rosmawati 2010). Although early detection of breast cancer can increase the survival rate, many women miss early detection due to lack of knowledge and information about breast health awareness (Avci 2008). Also, as the American Cancer Society has proven, if breast cancer is detected at an early stage (stage I–II), a 100–93 % survival rate can be achieved, and this rate decreases to 72–22 % if breast cancer is diagnosed at later stages (III–IV) (American Cancer Society 2013).

Many investigators have tried to determine the factors that affect women’s practice of BSE, because these factors are essential to plan effective intervention programs to improve BSE practice (Tarawneh and Attiyat 2013; Gumus et al. 2010). Current literature showed many barriers to BSE practice, which include social and cultural perceptions of breast cancer and breast self examination, socio-demographic factors, level of knowledge, and awareness (Hisham and Yip 2003; Rasu et al. 2011). Result of study done (Bit-Na et al. 2012) among Korean women shows (31.7 %) did not know how to do it, they would never have breast cancer (26.3 %), not effective for detecting breast cancer (17.1 %) and fear of detecting breast cancer (5.3 %). In Malaysia, there are few research done on the elimination of barriers of breast cancer awareness among young women (Hadi et al. 2010); because young women believe that they are not at risk of getting breast cancer (Johnson and Dickson-Swift 2008). The emergence of breast disease and the subsequent development of cancer tend to be more aggressive in young patients compared with breast cancer progressions in the older population (Johnson and Dickson-Swift 2008). Also, there is a lack of knowledge about the benefits of BSE among young women. For this reason, the aim of this study is to identify the barriers of BSE practice among undergraduate female students in Malaysia.

## Methods

### Study design and sampling method

A cross-sectional study was carried out among female undergraduate students in public universities in Klang Valley, Malaysia, between April and Jun 2012.

A multi-stage random sampling method was used to select students from public universities. A total of 810 students met the inclusion criteria and had gave informed consent to participate in the study. The inclusion criteria for this study was; age 20 years old and above, no history of breast cancer, and not pregnant or breastfeeding. This study obtained approval from the Ethical Committee of Universiti Putra Malaysia and the Ministry of Higher Education.

### Instrument

Data was collected via self-administrative a questionnaire which was developed by the researchers based on an extensive review of the literature. The content validity was evaluated by three expert from Community Health Department at Universiti Putra Malaysia to examine each item for congruence. The reliability of the questionnaire was determined by using test–retest reliability conducted among 80 female undergraduate students at Universiti Putra Malaysi not included in the study, and distributed in both English and Bahasa Malaysia language. The value of kappa for categorical data ranged between; breast cancer awareness (0.80–0.90), Breast cancer and BSE awareness (0.70–0.97). The value of intra-class correlation coefficient (ICC) for barriers of BSE practice (continuous data) was (0.70–0.80).

The questionnaire obtained information on respondents’ socio demographic characteristics, awareness of breast cancer and BSE, barriers for BSE practice, practice of BSE and source of information.

Socio demographic variables included: age, race, marital status, family income, family history of breast cancer (yes/no), personal history of breast disease (yes/no), hormonal drug usage (yes/no) and check the breast by doctor (yes/no).

Breast cancer and BSE awareness questions (yes/no response) included: “Have you ever heard about breast cancer?”, “Have you ever heard about BSE?”, “Do you know how to do BSE?”, and “What is the age of BSE?”.

The practice of BSE was assessed by asking respondents if they had ever done BSE (yes/no), and the frequency of doing BSE (once a month, occasionally and others).

The section about barriers for BSE practice had seven questions and respondents had to either “agree” or “disagree”.

### Analysis

All data was analyzed using PASW version 20.0 and a p less than 0.05 was considered as a level of significance. Descriptive statistics (mean and SD) were obtained for all the variables in the study. Chi-square analysis was used to determine the relation between categorical variables and BSE practice, and an independent sample t test was made for continuous data (age and family income).

## Results

### Respondent rate

A total of 810 female students were selected as the sample of the study. However, 68 respondents (8.3 %) refused to participate. The respondents’ rate derived in this study was 91.6 %.

### Socio-demographic characteristics of the respondents

The mean (SD) age of respondents was 21.7 (1.1) and ranged between 20 and 25 years old. The majority of them were Malay 709 (95.6 %), single 719 (96.9 %) and with monthly incomes of RM 4730.3 (2122.2). Family history of breast cancer was reported by 111 (15.0 %) of the respondents while 11 (1.5 %) had histories of breast problems (Table 1).

**Table 1:** Demographic and health characteristics of the participants (n = 742)

Variables	No	%
Age (mean ± SD)	21.7 ± 1.1	
Marital status		
Single	719	96.9
Others	23	3.1
Ethnicity		
Malay	709	95.6
Non-Malay	33	4.4
Family income (Mean ± SD)	4730.3 ± 2122.2*	
Family history of breast cancer		
Yes	130	16.4
No	662	83.6
Personal history of breast disease		
Yes	11	1.5
No	731	98.5
Hormonal drug usage		
Yes	40	5.1
No	752	94.9
Check your breast by doctor		
Yes	73	0.5
No	705	95.0

### Awareness of breast cancer and knowledge of BSE

Seven hundred thirty-eight (99.5 %) respondents have heard about breast cancer. Five hundred thirteen (69.1 %) had heard about BSE, but only 289 (38.9 %) respondents know how to do BSE. Regarding the age to begin BSE, only 303 (40.8) knew about it. Among those 513 who had heard about BSE, the main source of information was printed media (newspapers, brouchures) with 270 respondents (52.6 %), followed by information obtained from medical health personnel at 150 respondents (29.2 %), media at 60 respondents (11.7 %) and others at 33 respondents (6.4 %) (Table 2).

**Table 2:** Awareness of breast cancer and knowledge of BSE (n = 742)

Statements	Yes no (%)	No no (%)
Ever heard about breast cancer	738 (99.5)	4 (0.5)
Ever heard about BSE	513 (69.1)	229 (30.9)
Know how to do BSE	289 (38.9)	453 (61.1)
The age of BSE is starting from 20 years old	303 (40.8)	439 (59.2)
BSE is important for women to know how their breasts normally feel	260 (35.0)	482 (65.0)

### Practice of BSE

Although 738 (99.5 %) of the participants have heard about breast cancer, only 189 (25.5 %) performed BSE. Among those who practice BSE, most of them practice BSE occasionally at 96 respondents (50.8 %) and only 59 (31.2 %) respondents practice BSE once a month. The majority (50.2 %) stated the reason for performing BSE was to check their breast regularly, while 57 (30.1 %) had family history of breast cancer, 27 (14.2 %) had personal histories of breast diseases and 10 (5.2 %) had other reasons.

There were statistically significant relationships between those practicing BSE and those who did not practice BSE with age (*t* = −3.21, p = 0.001), marital status of respondents (χ^2^ = 8.91, p = 0.003) and personal history of breast disease (χ^2^ = 4.97, p = 0.02). There were no statistical differences between performing BSE with the rest of the variables.

### Barriers towards BSE practice

A total of 533 (74.5 %) respondents who did not practice BSE had nominated barriers for not performing it. The most common causes for not performing BSE were: I don’t know how to do it at 390 respondents (70.5 %); I don’t have any symptoms at 358 respondents (64.7 %); and I have worries in detecting breast cancer at 340 respondents (61.5 %). About 299 (54.1 %) respondents said that “doing BSE will take too much time” and 294 (53.2 %) respondents do not have enough privacy to do BSE (Table 3).

**Table 3:** Barriers towards BSE practice among respondents (n = 742)

Statements	No	%
I don’t know how to do it	390	70.5
Doing BSE will take too much time	299	54.1
I don’t have any symptoms	358	64.7
I am scared of being diagnosed with breast cancer	340	61.5
I don’t have enough privacy for BSE practice	294	53.2
I don’t feel it is necessary	317	57.3
BSE will be embarrassing to me	334	60.4

## Discussion

This study was conducted in order to examine BSE practice and barriers to BSE practice among Malaysian female students. In the current study, 69.1 % respondents were informed about breast self-examination. This is similar with the results of a previous study done in University of Gezira, Sudan (Abdelrahman and Yousif 2006) and inconsistent with the findings of a study from Malaysia (Al-Dubai et al. 2012) which found that 91 % of women were aware of breast self-examination. In this study, 189 (25.5 %) respondents perform BSE, but a small number of students (31.2 %) perform BSE once a month. The main reason for practicing BSE was to check their breast regularly. Similar to our findings, a study from Iran reported that 100 (26 %) women practice BSE, and most of them 53 (13.8 %) practice BSE occasionally (Akhtari-Zavare et al. 2014). This poor practice may be due to young women’s perceptions that they are healthy and thus do not need to perform a BSE.

Additionally, amongst this study samples, the most common source of information on BSE were printed media (newspapers, brochures) followed by information from medical health personnel. Similarly, in a study done by Al-Dubai et al. (2012) reported that newspapers and magazines were the main source of information on BSE. But, this finding is inconsistent with a previous study from Iran (Akhtari-Zavare et al. 2014) and Malaysia (Al-Naggar et al. 2011) who reported that television was the most common source of information.

Based on the results of this study, age, marital status and personal history of breast disease effect BSE practices (p < 0.05), but no association was found among those who practice BSE and the rest of the variables. This result is similar with the findings of other studies done by Redhwan et al. (2011) among female university students in Malaysia and Al-Azmy et al. (2013) among women attending primary health care clinics in Kuwait. There have been studies with conflicting results as well. While finding of studies done in Turkey (Doganer et al. 2014) and Kuwait (Al-Azmy et al. 2013) claimed that family history of breast cancer was related to performing BSE, another two studies revealed no significant relationship between BSE performance and family history of breast cancer (Tavafian et al. 2009).

In this study, the main reasons for not practicing BSE were “don’t know how to do BSE” (70.5 %); followed by “don’t have any symptoms” (64.7 %). Many young women believe that breast cancer affects only older women and thus, they are not at risk for getting breast cancer (Avci 2008). Also, lack of knowledge about how to do BSE among young Malaysian women have been due to inadequate education programs about breast health awareness for this target population. Consistent with our results, in many studies, women had mentioned that they don’t know how to do BSE (Redhwan et al. 2011; Tavafian et al. 2009) and they did not believe that they are at risk for getting breast cancer (Redhwan et al. 2011).

Others mention barriers towards breast self-examination practice such as “scared of being diagnosed with breast cancer” and “BSE will be embarrassing to me”. Similar to previous studies in Malaysia (Redhwan et al. 2011), Turkey (Avci 2008) and Iraq (Alwan et al. 2012) these fears may be a result of wrong perception of women on being diagnosed with breast cancer. Consequently, providing health educational programs about breast cancer and the significance of breast self-examination practices can reduce these worries due to wrong beliefs and motivate them to practice breast self-examination.

In the current study, “not enough privacy for BSE practice”, “not necessary” and “BSE takes too much time” were other barriers for performing BSE among young women in Malaysia. Providing breast health education programs among young women may help change the negative barriers towards breast self-examination and empower women to participate actively in making decisions regarding their health.

### Limitation of study

Since this study has been done among female undergraduate students, the findings cannot be generalized to the whole population in Malaysia.

### Implication for practice

Although there is no evidence that BSE lowers mortality from breast cancer, it should not be promoted to effectively detect breast cancer tumors in women. Women are at risk of harm from BSE including unnecessary breast biopsies, imaging tests and emotional duress (Hackshaw and Paul 2003).

Breast self-examination (BSE) might still be an important tool to improve breast awareness. Women are encouraged to take responsibility for their own health by taking opportunities such as during bathing or dressing to become familiar with their breasts at different times of the month and with age, looking and feeling for any changes from normal, and reporting any obvious changes promptly. Therefore, appropriate educational interventions are needed to encourage women to engage in regular breast awareness as well as to practice BSE (Bit-Na et al. 2003).

## Conclusion

In conclusion, the rate of BSE practice among young females in Malaysia is low and there are some barriers for doing BSE among this group. But as a this group is an educated group and has positive attitudes toward learning BSE from practice instructors, it is recommended to promote public breast health awareness campaigns through the media. Also, it is mandatory to establish or improve nationwide policy guidelines to increase the dissemination of information about breast cancer, the importance of breast self examination and other screening methods for controlling breast cancer among young females in Malaysia.
